# Structural Relaxation, Rejuvenation and Plasticity of Metallic Glasses: Microscopic Details from Anelastic Relaxation Spectra

**DOI:** 10.3390/ma16237444

**Published:** 2023-11-30

**Authors:** Michael Atzmon, Jong Doo Ju, Tianjiao Lei

**Affiliations:** 1 Department of Nuclear Engineering and Radiological Sciences, University of Michigan, Ann Arbor, MI 48109, USA; 2 Department of Materials Science and Engineering, University of Michigan, Ann Arbor, MI 48109, USA; 3 Materials Engineering, Testing and Standards (METS), Central Laboratory, Ford Motor Company, Dearborn, MI 48120, USA; 4 Department of Metallurgy and Materials Engineering, University of Alabama, Tuscaloosa, AL 35487, USA

**Keywords:** metallic glass, shear transformation zone, anelasticity, plasticity

## Abstract

The lack of periodicity and long-range order poses significant challenges in explaining and modeling the properties of metallic glasses. Conventional modeling of nonexponential relaxation with stretched exponents leads to inconsistencies and rarely offers information on microscopic properties. Instead, using quasi-static anelastic relaxation, we have obtained relaxation-time spectra over >10 orders of magnitude of time for several metallic glasses. The spectra enable us to examine in microscopic detail the distribution of shear transformation zones and their properties. They reveal an atomically-quantized hierarchy of shear transformation zones, providing insights into the effect of structural relaxation and rejuvenation, the origin of plasticity and the mechanisms of the alpha and beta relaxation.

## 1. Introduction

Amorphous solids, some of which form in natural processes, have been known to humans for thousands of years. Amorphous metallic alloys have only been known in recent decades. Initially formed by vapor deposition [1], they were later formed by solidification from the melt [2], resulting in metallic glasses. The first metallic glasses required high cooling rates to bypass crystallization, typically 10^6^ °C/s or higher, which limited at least one dimension to <10^−4^ m. A major breakthrough was achieved when new alloy compositions were discovered that required far lower cooling rates, resulting in bulk metallic glasses with dimensions that exceeded 10^−2^ m [3,4,5]. As a result, new, especially structural, applications became possible [6,7]. Additional experimental techniques became accessible, e.g., calorimetry in the supercooled liquid region and macroscopic mechanical testing, contributing to enhanced scientific understanding.

Scientists have long been intrigued and challenged by glasses, especially metallic glasses. The periodicity of crystalline solids allows for the use of powerful tools to measure and model their structure and properties. No such tools are available for amorphous solids. Furthermore, the structure and properties of metallic glasses depend strongly on their thermal history, since they relax structurally as they evolve toward a metastable equilibrium state. In addition, rejuvenation by thermal or mechanical means can reverse some of these processes. In equilibrium crystalline solids, in contrast, well-defined point- and extended defects can be introduced by thermal and mechanical treatment, but the base structure at atmospheric pressure is only a function of temperature. 

Dislocations, which play a central role in crystalline metal plasticity, are currently well understood [8]. The pioneering work of Sir William Bragg [9] with periodic, two-dimensional bubble rafts allowed him to visualize edge dislocations and their motion, and hypothesize their role in plasticity. Subsequent theoretical work led to a detailed understanding of their properties [8]. Crystal periodicity allows for imaging of dislocations in transmission electron microscopy [10].

In the absence of experimental and theoretical tools that parallel those available for crystalline solids, E. Orowan hypothesized that plastic deformation of disordered solids is accommodated by local, irreversible, rearrangements of clusters consisting of a few atoms/molecules [11]. Because of constraints posed by surrounding atoms/molecules, such rearrangements require thermal activation. Orowan proposed that if such rearrangements are rare and the matrix remains rigid, the memory of the rearranged domains is maintained, leading to anelasticity, i.e., time-dependent mechanical reversibility resulting from back-stress upon change in imposed constraints. On the other hand, if the rearranged domains exceed a threshold volume fraction, their memory is lost and permanent deformation, i.e., creep, results. This concept has also been incorporated into more-recent discussions [12,13,14]. 

Motivated by Bragg’s bubble-raft experiment and Orowan’s work, Argon and Kuo [15] created a two-dimensional physical analog of a binary amorphous solid by mixing bubbles of two sizes, each representing an atomic species, reflecting the ease of glass formation in a binary alloy compared to an elemental metal. This glass analog was then subjected to shear in its plane. While dislocations or point defects are readily visible in a periodic bubble raft, observing rearrangements in an amorphous bubble raft required careful tracking of the position of each bubble. At high stress, corresponding to low temperature, they observed local, disk-shaped, rearrangements. At low stress, corresponding to high temperature, they observed shear transformations of equiaxed bubble clusters, with shear values of the order of 0.2. Later experiments in colloidal glass were consistent with these observations [16]. Subsequent studies by numerous authors, e.g., Ref. [17], termed these clusters *shear transformation zones* (STZs). STZ behavior is the focus of the present review. The work included is based on our initial discovery of an atomically quantized hierarchy of STZs [18].

The present review consists of the following:

- A summary of Argon’s analysis of the mechanics and thermal activation of STZs.
- Our approach, which consists of (i) quasi-static anelastic recovery experiments that span more than ten orders of magnitude of time and (ii) computational determination of relaxation-time spectra by direct spectrum analysis (DSA).
- Relaxation-time spectra were determined numerically from the strain/time data. These provided valuable information on STZ size and property distribution, revealing an atomically-quantized hierarchy of STZs.
- Analysis of anelastic relaxation in the nonlinear regime, related to that of Argon and Shi’s creep experiments [19], provided an independent determination of the STZ transformation strain. Similar to the dislocation core in crystalline solids, this strain is far larger than the macroscopic yield strain.
- STZ spectra were computed from published dynamic-mechanical data. The results provide further, consistent, confirmation of the prior results and their analysis.
- Simple calculations show that stretched exponent fits, commonly used to fit non-exponential relaxation, are of limited utility. In particular, the time constant is ambiguous, and its apparent activation energy is not expected to reflect a specific physical process.
- The systematic error is evaluated for spectrum determination based on measurements conducted at discrete temperature increments and the assumption that the evolution at each temperature is dominated by a single activation free energy.
- Characterization of the details of structural relaxation and induced rejuvenation through their effect on STZ properties shows that these processes cannot be described with the evolution of a single variable.
- Anelastic relaxation spectra were obtained for La-based metallic glasses, some of which exhibit a distinct high-frequency/low-temperature (β) relaxation. Among the results, the following was found: contrary to suggestions by many authors, the α and β relaxation correspond to the same mechanism. Both are reversible when the corresponding STZs occupy a small volume fraction. The results also suggest that different elements are involved in slow vs. fast STZs, corresponding to the α and β relaxation, respectively. Simulations of dynamic-mechanical behavior for experimentally obtained STZ spectra further support the notion that the α and β relaxation correspond to the same mechanism. That curves obtained at different temperature can be shifted into a single master curve cannot be seen as proof of a single activation energy.
- By comparing metallic glasses that exhibit different degrees of plasticity at similar composition, plasticity is explained in terms of the volume fraction occupied by kinetically active *potential* STZs.

## 2. Theory of Thermally-Activated Shear Transformation

While STZ analyses in the literature are typically based on an assumption of a single STZ size, our observations, reviewed below, indicate a spectrum of sizes and properties. We therefore modified Argon’s kinetic model [12] to express the shear strain rate as a function of shear stress *σ_s_* to account for a spectrum of STZ types, indexed initially with *m*, each contributing additively to the total shear strain rate [18]:

(1) $${\dot{\gamma }}_{m}=2c_{m}\gamma_{o}^{c}\nu_{G}exp\left(-\frac{∆F_{m}}{kT}\right)sinh\left(\frac{{\sigma_{s}\gamma }_{o}^{T}\Omega_{m}}{2kT}\right),$$

where $\gamma_{0}^{T}$ is the transformation shear strain of an STZ unconstrained by the surrounding matrix (≈0.2 [18]), and $\gamma_{0}^{c}=\left[2\left(4-5\nu \right)/15\left(1-\nu \right)\right]\gamma_{0}^{T}$ is the constrained value with ν=0.324 [20] being Poisson’s ratio. $\nu_{G}$ is the attempt frequency, *k* is the Boltzmann constant, and *T* is the temperature. $\Omega_{m}$ is the *m*-type STZ volume, so $\gamma_{o}^{T}\Omega_{m}$ is the activation volume. $∆F_{m}$ is the activation free energy for the shear transformation of *m*-type STZs [18,19]:

(2) $${∆F}_{m}=\left[\left(\frac{\left(7-5\nu \right)}{30\left(1-\nu \right)}+\frac{2\left(1+\nu \right)}{9\left(1-\nu \right)}{\bar{\beta }}^{2}\right)\gamma_{0}^{T}+\frac{1}{2}\frac{\bar{\sigma_{STZ}}}{\mu }\right]\mu \gamma_{0}^{T}\Omega_{m},$$

where the term with ${\bar{\beta }}^{2}\;(∼1)$ accounts for the dilatation associated with a shear transformation. $\bar{\sigma_{STZ}}$ is the shear resistance of STZs, μ is the shear modulus, and $\;\bar{\sigma_{STZ}}/\mu =0.025$ [21]. This third term in the brackets is negligible compared to the first and second term. Note that in Ref. [12], the pre-exponential factor *c* is interpreted as the volume fraction occupied by *potential* (or *fertile*) STZs, i.e., atomic clusters capable of undergoing a shear transformation. In the present work, the *c_m_* are resolved by STZ type, *m*, and obtained from experiment, as shown below. It is noted that in the notation used, overlapping *potential* STZs are counted multiple times. Equation (1) is valid as long as only a small fraction of them undergoes shear transformations.

## 3. Experiments and Spectrum Determination

**Figure 1:**
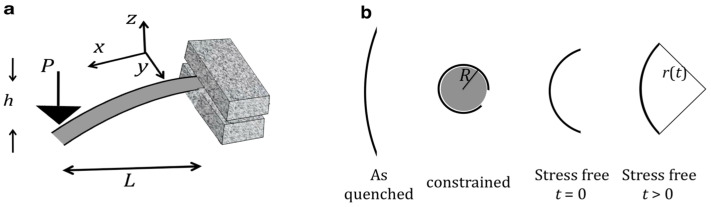
Measurement techniques. (**a**) Cantilever method. The displacement *h* is monitored as a function of time at a fixed load, *P*. The instantaneous displacement is the elastic component; (**b**) Mandrel method. The sample was constrained for 2 × 10^6^ s at varying radii, after which the radius of curvature was monitored as a function of time in a stress-free condition. Reproduced from Ju, J.D.; Jang, D., Nwankpa, A; Atzmon. M. An atomically quantized hierarchy of shear transformation zones in a metallic glass. *J. Appl. Phys*. **2011**, 109. with permission of AIP Publishing [18].

The experimental basis for the presently reviewed work is the room-temperature measurement of quasi-static anelastic relaxation (Figure 1) over a wide range of time constants. The simple exponential decay for each time constant in the spectrum facilitates the data analysis, as compared with commonly used dynamic-mechanical analysis (discussed below). For short time constants, ~1.5 × 10^−3^–200 s, using a nanoindenter at fixed force to monitor the displacement of a cantilever (Figure 1a) as a function of time provided the strain evolution. For long time constants, up to ~6 × 10^7^ s, instrumented measurements pose stability challenges. Instead, therefore, 20–40 μm thick ribbon samples were constrained for 2 × 10^6^ s around a mandrel at a fixed radius of curvature; subsequently, their radii of curvature were monitored as a function of time in a stress-free state. Except for the early study of Al_86.8_Ni_3.7_Y_9.5_ [18], the sample curvature determination was performed using an automated fit to its image. Based on the confirmed linearity of the relaxation process, the strain and stress at any distance from the neutral midplane were calculated as a function of time. The strain at the surface is used in all reported data.

While knowledge of the spectrum allows for an explicit expression of the strain evolution, the reverse is not true – the spectrum is only implicitly determined. Therefore, the spectra needed to be determined numerically, using what is known as direct spectrum analysis [22]. From the strain(time) data, relaxation-time spectra were computed using CONTIN, a portable package for such inverse problems [23,24,25]. This software applies a penalty to rapid variations in the solution, thus eliminating unphysical results. The following strain(time) functions were fitted to $\varepsilon_{an}\left(t\right),$ the anelastic time-dependent strain, based on a linear solid model (see below): (a) For the mandrel experiment, $\varepsilon_{an}\left(t\right)/\varepsilon_{el}^{0}=c_{\infty }+\sum_{i=1}^{N_{1}}\varepsilon_{i}exp\left(-t/\tau_{i}\right)$, where $c_{\infty }$ and the $\varepsilon_{i}$ are fitting parameters. The former represents possible processes with time constants significantly greater than the duration of the experiment, and the $\varepsilon_{i}$ are a discrete best estimate of the continuous spectrum sought. $\varepsilon_{el}^{0}$ is the elastic strain at the end of the constraining period. The time constants $\tau_{i}$, for which the spectrum $\varepsilon_{i}$ is defined, are logarithmically spaced because of the wide range of measurement times. (b) For the cantilever experiment, the function fitted was $\varepsilon_{an}\left(t\right)/\varepsilon_{el}^{0}=c_{\infty }+Bt+\sum_{i=1}^{N_{2}}\varepsilon_{i}exp(-t/\tau_{i})$, where the $\tau_{i}$ and $\varepsilon_{i}$ play the same role as for the mandrel experiment, and *B* is a constant that approximates the rates of the wide range of processes with time constant greater than the duration of the cantilever measurement (200 s). Up to ~50 time points, *N_1_* and *N_2_*, were used as an approximation of a continuum spectrum, fewer than the number of data points in order to avoid overdetermination. Extensive tests were conducted with simulated data corresponding to assumed input spectra, with added noise, verifying that these spectra can be recovered by the fitting procedure.

## 4. An Atomically Quantized Hierarchy of STZs [18]

**Figure 2:**
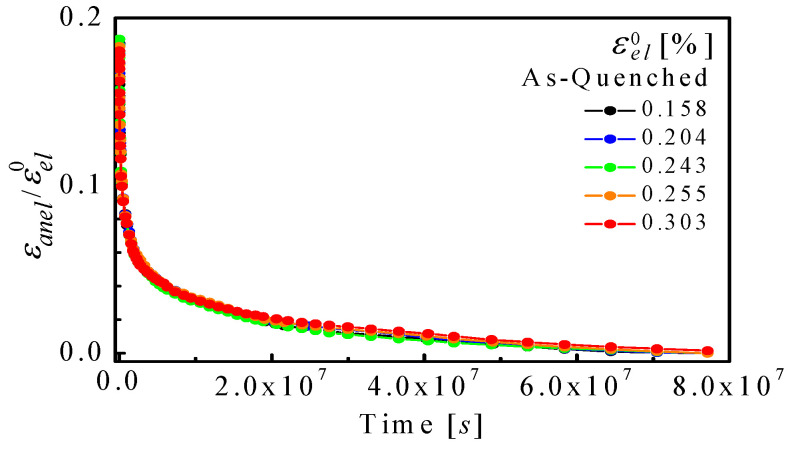
Al_86.8_Ni_3.7_Y_9.5_: Anelastic strain evolution following equilibration at different mandrel radii. The strain is normalized by the elastic strain at equilibrium, prior to removal of the constraint. Reproduced from Ju, J.D.; Jang, D., Nwankpa, A; Atzmon. M. An atomically quantized hierarchy of shear transformation zones in a metallic glass. *J. Appl. Phys*. **2011**, 109. with permission of AIP Publishing [18].

**Figure 3:**
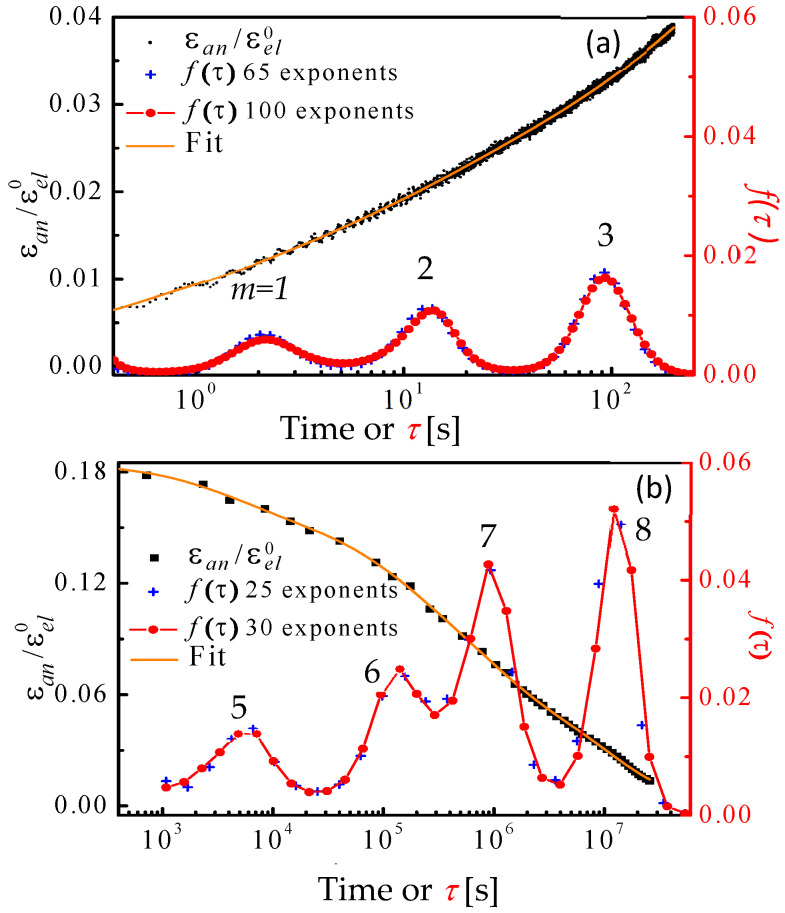
Al_86.8_Ni_3.7_Y_9.5_: Sample relaxation curves and corresponding relaxation-time spectra. (**a**) Cantilever measurement, performed at fixed load, *P* = 0.2 mN, i.e., fixed stress. (**b**) Mandrel measurement, performed in a stress-free condition after equilibration under constraint. For each case, two spectra, *f*(*τ*), are shown, obtained from fits with different numbers of fitting parameters. Reproduced from Ju, J.D.; Jang, D., Nwankpa, A; Atzmon. M. An atomically quantized hierarchy of shear transformation zones in a metallic glass. *J. Appl. Phys*. **2011**, 109. with permission of AIP Publishing [18].

These original experiments were performed with Al_86.8_Ni_3.7_Y_9.5_ metallic glass ribbons. For the mandrel experiments, the anelastic strain, $\varepsilon_{anel}\left(t\right)$, was monitored at room temperature after constraint removal as a function of time for ~8 × 10^7^ s, as it recovered its original shape. It is shown in Figure 2, normalized by $\varepsilon_{el}^{0}$ for several mandrel radii used to constrain the samples. All curves coincide, indicating that the anelastic processes are in the linear regime. This directly supports the assumption that the strain profile across the sample thickness is linear. It also implies that no significant yield had taken place. Visual inspection reveals that multiple time constants govern the anelastic recovery. Figure 3 shows representative $\varepsilon_{an}\left(t\right)$/$\varepsilon_{el}^{0}$ curves, along with corresponding computed spectra, f(τ), for the cantilever (Figure 3a) and mandrel (Figure 3b) experiments. Fits obtained with different numbers of fitting points, *N_1_* and *N_2_*, demonstrate the consistency of spectrum computation.

**Figure 4:**
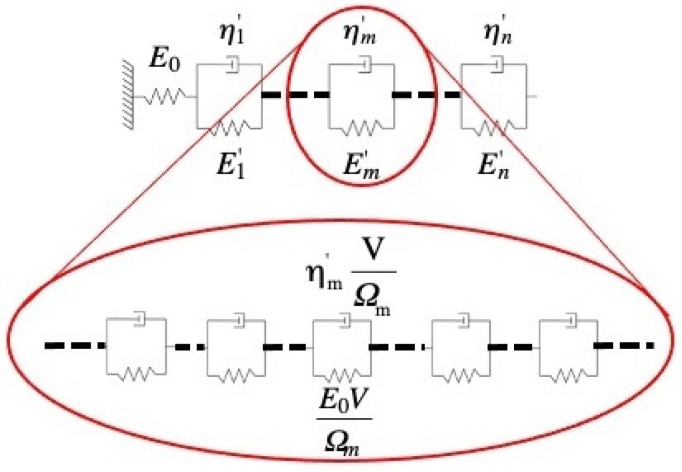
Top: linear solid model: *n* anelastic processes act in series, each represented by a Voigt unit. *m*-type sites are associated with Young’s modulus of $E_{m}^{\prime }$ and viscosity $\eta_{m}^{\prime }$, both effective quantities that are inversely proportional to the volume fraction of these sites. *E_0_* is the high-frequency Young’s modulus. Bottom: illustration of the contribution of each *m*-type STZ to Voigt Unit *m*. Reproduced from Ju, J.D.; Jang, D., Nwankpa, A; Atzmon. M. An atomically quantized hierarchy of shear transformation zones in a metallic glass. *J. Appl. Phys*. **2011**, 109. with permission of AIP Publishing [18].

Surprisingly, the spectra in Figure 3 consist of distinct peaks, indexed with *m*. This discovery motivated modeling the metallic glass as a linear solid [26], consisting of a spring (effective high-frequency Young’s modulus *E_0_*), representing elastic behavior, in series with a series of Voigt units, each consisting of a spring (effective modulus $E_{m}^{\prime }$) and dashpot (effective viscosity $\eta_{m}^{\prime }$) in parallel (Figure 4, top). Under zero or fixed stress, each Voigt unit relaxes exponentially with a time constant

(3) $$\tau_{m}=\frac{3\eta_{m}^{\prime }}{E_{m}^{\prime }}\;,$$

where the factor of 3 accounts for the conversion of uniaxial to shear viscosity. The use of linear viscosity is valid when the sinh term in Equation (1) is linear in the stress, as confirmed by Figure 2 [18]. At the end of the constraining period, the Voigt units with a time constant shorter than the constraining time reach mechanical equilibrium with each other and the spring that represents the elastic behavior, yielding

(4) $$E_{m}^{\prime }=\frac{\varepsilon_{el}^{0}}{\varepsilon_{m}^{0}}E_{0},$$

where *E_0_* (see Figure 4) is the sample’s high-frequency Young’s modulus. By definition,

(5) $$\varepsilon_{m}^{0}=\varepsilon_{el}^{0}\times \int_{m}^{}f(\tau )dln\tau ,$$

is the contribution of Voigt Unit *m* to the strain, with integration over peak *m* in the spectrum. It is noted that in the spectrum in Figure 3b, the area under peak *m* = 8 does not reflect mechanical equilibrium because the corresponding time constant is longer than the constraining time.

The centroids of the spectrum peaks in Figure 3 are the time constants $\tau_{m}$, and their areas yield $E_{m}^{\prime }$ according to Equation (4). Then, using Equation (3), the $\eta_{m}^{\prime }$ values are obtained. Now the results can be related to the constitutive law (Equation (1)) using the definition of linear viscosity ${\dot{\gamma }}_{m}=\frac{\sigma_{s}}{\eta_{m}^{\prime }}$, where *σ_s_* is the net shear stress on the dashpot in Unit *m*. Straightforward algebra [18] yields a simple expression for *c_m_*:

(6) $$c_{m}=\frac{\varepsilon_{m}^{0}}{\varepsilon_{el}^{0}}=\int_{m}^{}f(\tau )dln\tau .$$

The lower part of Figure 4 illustrates the contribution of each individual type-*m potential* STZ to Voigt Unit *m* in the upper part. 

**Figure 5:**
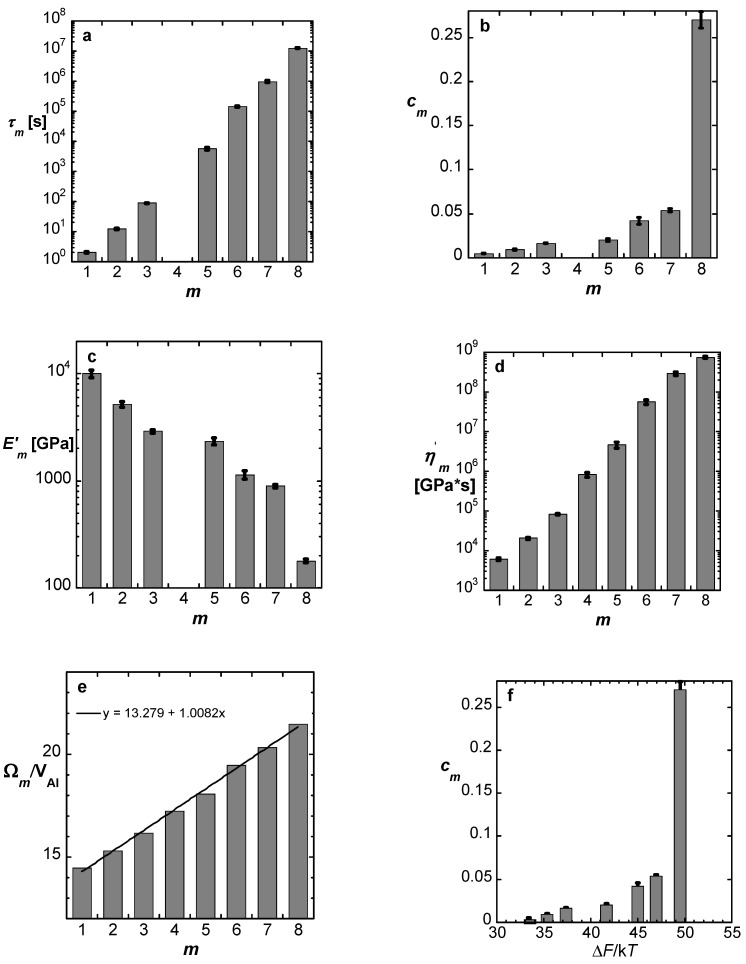
Al_86.8_Ni_3.7_Y_9.5_: Calculated properties of the respective anelastic processes *m* = 1–8. (**a**) Time constants. (**b**) Volume fraction of *potential* STZs. (**c**) Effective macroscopic Young’s modulus. (**d**) Effective macroscopic viscosity. (**e**) STZ volume in units of atomic volume of Al, V_Al_ = 16.6 × 10^−30^ m^3^. Values for *m* = 4 were obtained by interpolation. (**f**) Volume fraction of *potential* STZ as a function of Δ*F*/k*T*. The error bars are the standard deviation of the mean, obtained by averaging over multiple measurements. Reproduced from Ju, J.D.; Jang, D., Nwankpa, A; Atzmon. M. An atomically quantized hierarchy of shear transformation zones in a metallic glass. *J. Appl. Phys*. **2011**, 109. with permission of AIP Publishing [18].

Based on the equations above, with linearized sinh in Equation (1), the properties of each *potential* STZ type, *m*, are plotted in Figure 5. Using literature data for the elastic constants, the volume of type-*m* STZs, *Ω_m_,* is obtained and displayed, normalized by the atomic volume of Al, in Figure 5e. The error in *Ω_m_* is small since it appears in the exponent in Equation (1). Somewhat fortuitously, the slope of this plot is within < 1% of 1. This one-atom increment in *Ω_m_* leads to the conclusion that the peaks in the spectrum represent an *atomically-quantized hierarchy of STZs* — the spectrum peaks correspond to STZs that consist of *n* = 14,…, 21 atoms. The dominance of a single element, Al, likely facilitates the resolution of this hierarchy. The activation free energies corresponding to this STZ hierarchy, ${∆F}_{n}$, range from 0.85 to 1.26 eV (Equation (2), Figure 5f).

Later experiments [27], conducted at longer duration (constraining time 4.4 × 10^6^ s and anelastic recovery for 1.1 × 10^8^ s) further confirmed the hierarchy, showing the signature of STZs consisting of 22 atoms. The *c_n_* obtained allowed for modeling the size-density distribution. It was assumed that a cluster needed to contain a sufficient amount of free volume, >v^*^, in order for it to be capable of a shear transformation. Using Poisson statistics for the free-volume distribution, the best fit to the data was obtained when this threshold, v^*^, varied only slightly with size, as *n^0.22^* [27]. This weak dependence is expected if free volume is shared dynamically within the STZ on a time scale required for shear transformation.

## 5. The Transformation Strain [28]

**Figure 6:**
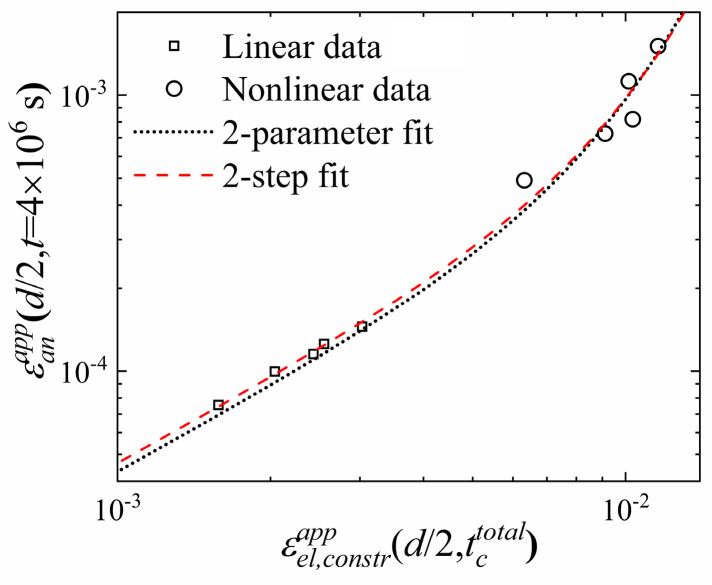
Al_86.8_Ni_3.7_Y_9.5_: Apparent anelastic strain after unconstrained relaxation for *t* = 4 × 10^6^ s as a function of the apparent elastic strain at the end of the constraining period for varying constraining radii. Both are computed for the sample surface from the curvature. Each symbol represents one sample. Linear data are from Ref. [18]. Deviation from linearity occurs at high strain. Comparison between the two-parameter fit (dotted line) and two-step fit (dashed line) (see Ref. [28]): the latter yields a better fit for the small-strain data than the former. Reproduced from Lei, T.J.; Atzmon, M. Activation volume details from nonlinear anelastic deformation of a metallic glass. *J. Appl. Phys.* **2019**, 126, 185104, with permission of AIP Publishing.

The activation volume for shear transformation is the product of the transformation strain $\gamma_{0}^{T}$ and STZ volume *Ω_m_*. They cannot be determined independently from the data above because only the product ${({\gamma_{0}^{T})}^{2}\Omega }_{m}$ appears in the (linearized) sinh term in Equation (1) and in Equation (2), recalling that the third term in Equation (2) is negligible. Therefore, $\gamma_{0}^{T}$ had been estimated from experiments conducted in colloidal glass [16] and from molecular dynamics simulations [29,30], $\gamma_{0}^{T}\approx 0.2$, which affects the resulting values of *Ω_m_*. An independent determination of $\gamma_{0}^{T}$ requires experiments in the nonlinear regime of the sinh term of Equation (1). Such analysis was carried out by Argon and Shi [19] for nonlinear creep data. To complement the linear results presented above, we conducted nonlinear anelastic relaxation experiments on Al_86.8_Ni_3.7_Y_9.5_ metallic glass ribbons by using smaller mandrel diameters, 0.35 to 0.49 cm, resulting in bending strain values up to 0.0155, compared with 0.00303 for the prior experiments in the linear regime. For the resulting stress, the sinh term in Equation (1) is nonlinear. The volume fraction occupied by STZs is still small, ≤7.2%, so that STZ interactions are negligible and the STZs can be considered isolated. Yield was ruled out by verifying the absence of change in the sample geometry following brief constraint. The normalized, apparent, anelastic strain, determined from the stress-free curvature at *t* = 4 × 10^6^ s after the release of the constraint, is shown as a function of the elastic constraining strain in Figure 6, for both the nonlinear and earlier linear data. At such a point in time, the fast STZs have relaxed, and the largest STZs activated, with *n* = 21, dominate the relaxation behavior. 

Fitting the nonlinear equations to these data, $\gamma_{0}^{T}$ = 0.17 is obtained. By computing the fit sensitivity to this value, the random error is determined to be ±3%. As before for the *Ω_m_*, this small error is due to the fact that $\gamma_{0}^{T}$ appears in the exponent in Equation (2). The value obtained, $\gamma_{0}^{T}$ = 0.17, is reasonably close to the value assumed in the analysis in Ref. [18], 0.2, as summarized above. It is much greater than the universal, low-temperature, macroscopic yield strain observed in metallic glasses, 0.036 [31]. An important parallel to crystalline solids helps illustrate this difference in magnitude: the strain in a dislocation core is of the order of 1, yet the yield strain in metals is below 0.01. It is worth noting that in some studies, equating the transformation strain to the yield strain resulted in unphysically large STZ sizes being backed out from the data [32,33].

## 6. Dynamic-Mechanical Analysis [34]

In the analysis of quasi-static data obtained at room temperature, the temperature dependence of the strain rate had to be assumed, see Equations (1) and (2). Measurements at varying temperature involve stability challenges because of the long time involved. While fitting frequency-dependent dynamic-mechanical data poses challenges, it enabled us to carry out a direct evaluation of the temperature dependence. 

The analysis methodology to be used was evaluated by simulating the loss modulus [26], $E_{s}^{\prime \prime }\left(\omega \right),$ as a function of frequency *ω* for an input spectrum of time constants, $f^{a}\left(\tau \right),$ based on Ref. [18]:

(7) $$E_{s}^{\prime \prime }(\omega )=E_{0}^{\prime \prime }\times \int f^{a}(\tau )\frac{{\omega \tau }_{i}}{1+{\left({\omega \tau }_{i}\right)}^{2}}dln\tau .$$

$E_{s}^{\prime \prime }\left(\omega \right),$ plus added noise, was then fitted with

(8) $$E\prime \prime (\omega )=\sum_{i=1}^{N}f_{i}\frac{{\omega \tau }_{i}}{1+{\left({\omega \tau }_{i}\right)}^{2}},$$

where the time constants $\tau_{i}$ are logarithmically spaced, *N* = 70 and the $f_{i}$ are fitting parameters representing a discrete best estimate of the spectrum. Iterative fits were repeated for increasingly tighter target tolerance values [34] for each of the multiple simulated spectra. It was found that input spectra were most-accurately recovered for the tolerance value at which *R^2^*, the coefficient of determination, began to increase. This tolerance value was used as the best-fit criterion when analyzing the experimental data.

**Figure 7:**
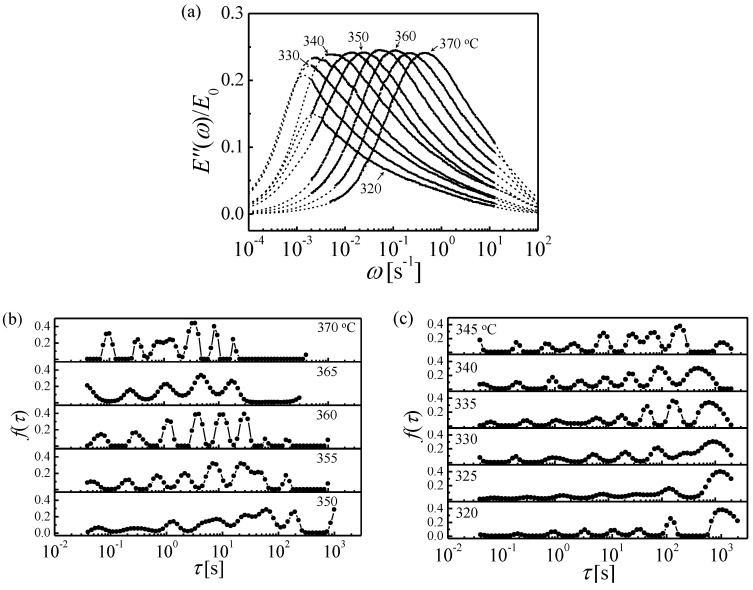
Zr_46.8_Ti_13.8_Cu_12.5_Ni_10_Be_27.5_: (**a**) Digitized loss moduli [35] with DSA fits. (**b**,**c**) Spectra obtained from these fits above and below *T_g_*. Reprinted from Ju, J.D.; Atzmon, M. A comprehensive atomistic analysis of the experimental dynamic-mechanical response of a metallic glass. *Acta Mater.* **2014**, *74*, 183–188, Copyright (2014), with permission from Elsevier [34].

**Figure 8:**
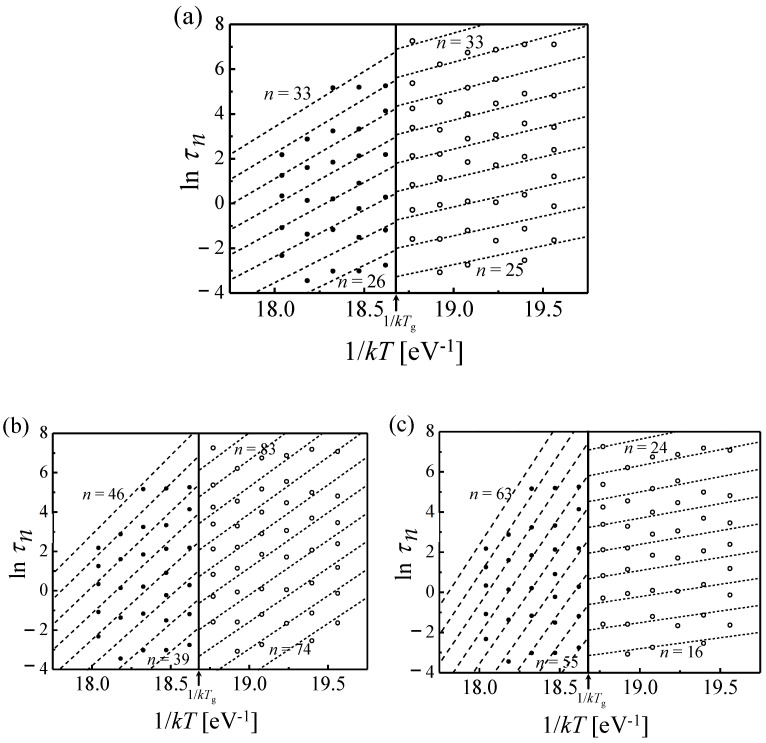
Zr_46.8_Ti_13.8_Cu_12.5_Ni_10_Be_27.5_: ln*τ*, determined from the median of the respective peak in the relaxation-time spectra of Figure 7, plotted as a function of 1/*kT* for three tentative groupings of *τ_n_* (**a**–**c**) below (gray circles) and above *T_g_* (black circles). Simultaneous fit performed using Equation (9) is shown with dashed lines for each *n*. Above *T_g_*, linear temperature dependence of the modulus was used. Out of nine possible combinations, continuity of the fits and *n* at *T_g_* is obtained only for the combination displayed in (a). Reprinted from Ju, J.D.; Atzmon, M. A comprehensive atomistic analysis of the experimental dynamic-mechanical response of a metallic glass. *Acta Mater.* **2014**, *74*, 183–188, Copyright (2014), with permission from Elsevier [34].

Because of the steep variation of the loss modulus with temperature, curves measured as a function of frequency, acquired at multiple temperatures, lend themselves better to fitting the model than the more common curves obtained as a function of temperature. Therefore, the extensive data of Ref. [35], obtained for Zr_46.8_Ti_13.8_Cu_12.5_Ni_10_Be_27.5_, were used in the analysis. The fits and corresponding spectra are shown in Figure 7. The time constants obtained from each peak are shown in Arrhenius plots in Figure 8 as a function of temperature. The goal was to obtain simultaneous fit lines for all STZ sizes, based on an atomically quantized hierarchy of STZs. For each trial STZ size *n*, the time constant was expressed as a function of temperature based on the theory reviewed above:

(9) $$\tau_{n}=\frac{3kT}{2\mu (1+\nu )\nu_{G}{\gamma_{o}^{c}\gamma }_{0}^{T}\Omega_{n}}exp\left(\frac{∆F_{n}}{kT}\right),$$

with ${∆F}_{n}$ given by Equation (2). 

Since the data [35] were obtained both below and above the glass transition temperature, *T_g_*, and the shear modulus varies significantly with temperature in the latter range, its approximate linear temperature dependence above *T_g_* was included in the fits [36,37,38,39]. They were carried out simultaneously for all values of *T* and *n*. The main challenge was determining which set of data points corresponded to the same STZ size, *n*, within a multi-*n* simultaneous fit. Several plausible groupings were attempted each below and above *T_g_*. The only combination of such sets that yields continuity and the same *n* values across *T_g_* is that shown in Figure 8a. The resulting *n* values range from 25 to 33, with corresponding activation free energies of 1.75–2.3 eV. These results are consistent with those of Ref. [18], further confirming them and the model used. These higher values of *n*, compared with 14–22 at room temperature in Ref. [18], are expected since the spectra increase monotonically and larger STZs become active with increasing temperature. 

**Figure 9:**
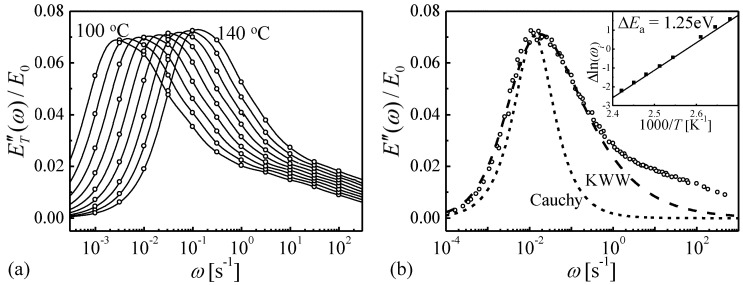
(**a**) $E_{T}^{\prime \prime }(\omega )$, loss modulus, calculated at *T_i_* steps of 5 K from the spectrum obtained in Ref. [18]. (**b**) Master curve obtained by shifting sets of points obtained at each *T_i_* to coincide with the curve at $T_{ref}$ = 388 K. A Cauchy function, corresponding to a single activated process, and a KWW fit to the main part of the curve, are included. Inset: Arrhenius plot of the shift factor as a function of reciprocal temperature. Reproduced from Ju J.D.; Atzmon, M. Atomistic interpretation of the dynamic response of glasses. *MRS Comm.* **2014**, 4, 63–66 with permission from SNCSC [44].

It is instructive to evaluate at this point the time-temperature superposition principle [40,41,42,43]. One of its formulations is that for a process with a single activation energy $∆E_{a}$, loss modulus curves measured as a function of frequency, *E″*(*ω*), shifted on a logarithmic scale by

(10) $$∆\ln\left(\omega_{i}\right)=\frac{∆E_{a}}{kT}\left(\frac{1}{T_{i}}-\frac{1}{T_{ref}}\right)$$

will coincide in a single master curve with the curve measured at the reference temperature $T_{ref}$. Using these required shift values for a set of measured *E″*(*ω*) curves as a function of temperature, an apparent activation energy can be obtained. In Ref. [44], we used the spectra obtained in Ref. [18] to calculate the corresponding *E″*(*ω*) curves at several temperatures, $T_{i}$, based on Equations (7) and (9), as shown in Figure 9a. These were then shifted to obtain a master curve, as shown in Figure 9b. An Arrhenius plot of $∆\ln\left(\omega_{i}\right)$ (Figure 9b, inset) yielded an activation energy of 1.25 eV, despite the fact that the input STZ spectrum contributing to *E″*(*ω*) ranged from 0.85 to 1.26 eV. The 1.25 eV value obtained reflects the dominance of the largest STZs, for which the concentration of corresponding *potential* STZs is the highest [18]. This leads to the important conclusion that observed Arrhenius behavior of the shift that yields a master curve may be insufficiently sensitive to rule out a spectrum of activation energies.

## 7. The Stretched Exponent [45]

**Figure 10:**
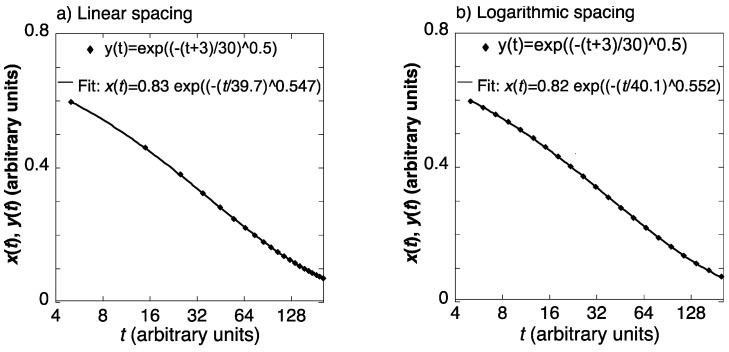
A hypothetical time-dependent quantity, described as a stretched exponent with a small shift (y(*t),* open circles) is fitted with an unshifted stretched exponent (*x*(*t*), Equation (1), line). (**a**) Linearly spaced time points; (**b**) Logarithmically spaced time points. Significantly different *τ* and *β* are obtained. Reproduced from Atzmon, M. The pitfalls of empirical fitting of glass relaxation data with stretched exponents. *J. Appl. Phys.* 2018, 123, 065103, with the permission of AIP Publishing [45].

This section further rationalizes the need to compute relaxation-time spectra from the anelastic relaxation data described above. Many processes in nature exhibit exponential decay, which takes place when the rate of change of a variable is proportional to the variable itself. However, one often encounters deviations from this ideal behavior. Early on, Kohlrausch [46] proposed describing the electrostatic discharge of a capacitor as a function of time with a stretched exponent,

(11) xt=x0exp−tτsβ,

with $\tau_{s}$ and *β* being constants and *x*(0) being the initial charge. Currently, many studies of non-exponential relaxation in disordered materials employ this expression, referred to as Kohlrausch-William Watts (KWW) [47,48]. This time dependence has also been used to derive the behavior in the frequency domain [47]. The expression, which often provides good fits, is phenomenological in most cases, with few exceptions for which it results from a mechanistic model [49,50,51], usually near or above the glass transition. Despite the phenomenological nature of Equation (11), it is often assumed to represent a physical process [52,53,54,55,56,57,58,59,60], leading to conclusions that are difficult to support. Examples among these are the KWW fitting of the dielectric loss or the loss modulus in glass. Deviations from the fitted KWW curve at high frequency, also seen in our *E″*(*ω*) calculated from experimental spectra, are interpreted by some authors as resulting from a separate relaxation mechanism. This amounts to assuming a priori that the behavior should correspond to the spectrum of time constants consistent with KWW behavior. However, as our present results and analysis show, a single mechanism, namely shear transformations, can explain the behavior without relying on this often unsupported restriction. The very interpretation of $\tau_{s}$ as a time constant is problematic because of an internal inconsistency: simulated data points, based on a stretched exponent, shifted by 10% of $\tau_{s}$, exp(−((*t*+3)/30)^0.5^), were fitted with an unshifted stretched exponent (Figure 10). The fitting parameters depend on the range of *t* values and the manner in which the points are spaced on the *t* axis. However, as shown in Figure 10, similar results are obtained for linear (a) and logarithmic (b) spacing, where the former gives greater weight to long time values. Both yield good fits with similar fitting parameters. Remarkably, the $\tau_{s}$ values obtained are higher by >30% than the value of 30 used to simulate the data points. This is a result of the fact that, unlike for a simple exponent, the relative rate of change of the stretched exponent is not constant in time. The common assumption that the temperature dependence of $\tau_{s}$, however obtained, can yield an activation energy [53,57] is therefore not supported. For these reasons, the presently reviewed work is based on spectrum determination from the data without prior assumptions.

## 8. Systematic Error in Spectrum Determination by Temperature Stepping [61] 

**Figure 11:**
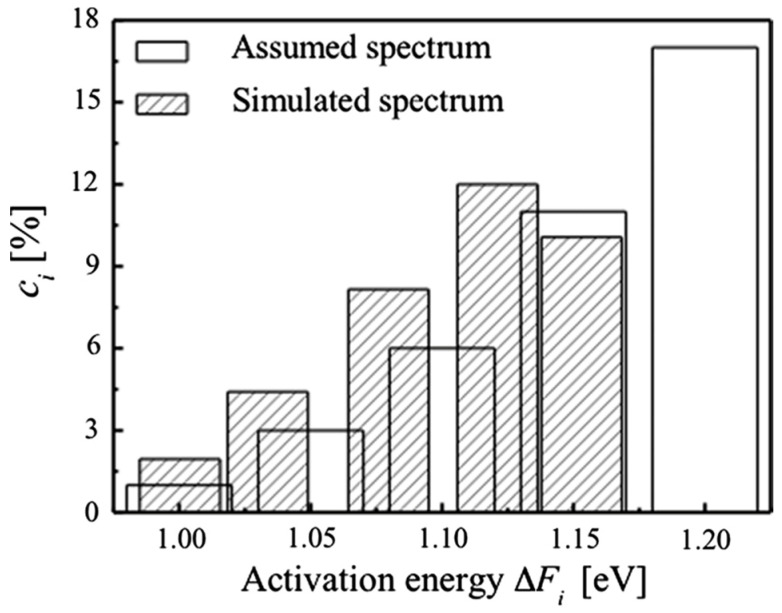
Assumed spectrum compared with that calculated from it using the temperature stepping approximation. Both the activation-energy values and spectrum shape are affected. The latter spectrum is similar to those in Ref. [62]. Reprinted from Ju, J.D.; Atzmon, M. Evaluation of approximate measurements of activation-free-energy spectra of shear transformation zones in metallic glasses, *J. Alloys Comp.* **2015**, 643, S8–S10, Copyright (2014), with permission from Elsevier [61].

One method of obtaining approximate spectra from relaxation measurements is based on measurements conducted by stepping the temperature from the lowest to the highest. It is then assumed that the behavior at each step *i* at temperature *T_i_*, is dominated by a single activation free energy given by ∆Fi=−k∂lnγ˙∂(1T)Ti. The assumption implicit in this approximation is that at each step, processes with lower activation free energy have equilibrated, while those with higher activation free energy are frozen. Argon and Kuo [62] proposed this method to evaluate the activation free energy spectrum for torsional creep experiments. One aspect of the spectrum they obtained was a drop at the highest value of $∆F_{i}$. In contrast, Refs. [18,27] exhibit a monotonically increasing spectrum, which is also consistent with the free-volume model [27]. In this context, it is instructive to assess the error introduced by the approximation of a dominant activation free energy at each temperature step. For this purpose, we assumed a simple, monotonic, spectrum of activation free energies, qualitatively similar to that in Figure 5f. By simulating the process of anelastic relaxation at stepwise increasing temperatures, we obtained a simulated, apparent spectrum, based on the approximation of Ref. [62], which exhibits a decrease at the highest activation free energy (Figure 11). Comparison with the assumed input spectrum illustrates that the observed decrease is an artifact of the temperature-stepping method: processes with high activation energy are not completely frozen at lower temperatures, thus reducing their apparent contribution. Their participation at lower temperature also explains the shift to lower activation energies, seen in Figure 11.

## 9. Characterization of Structural Evolution [63]

As mentioned in the introduction, glasses, unlike crystalline solids, undergo continuous evolution toward an internal equilibrium state. Below the glass transition temperature, this state is typically not reached on practical time scales. This structural relaxation leads to a small increase in the elastic moduli and decrease in the stored enthalpy and electrical conductivity. Atomic transport rates can decrease by orders of magnitude, and with them the rates of processes such as creep and diffusion. Embrittlement often results. Zhao et al. [64] recently reported an example in which the friction coefficient increases with relaxation while the wear volume decreases. The degree of property change does not evolve linearly in the degree of relaxation: as Kumar et al. report [65], embrittlement can occur rapidly during the initial stages of relaxation, where the latter is characterized by calorimetry. Efforts to design tough metallic glasses have included inducing the process opposite to structural relaxation, namely rejuvenation. This has been accomplished, e.g., by annealing above *T_g_* [65] or by plastic deformation, including shot peening [66]. In addition, cyclic elastic loading [67], constrained loading [68] and irradiation [69,70] have led to rejuvenation. Cycling between room and cryogenic temperature has also been reported to lead to rejuvenation [71], as determined from measurements of stored enthalpy and yield. The authors proposed a rejuvenation mechanism due to heterogeneity of the thermal expansion coefficient, leading to microscopic stresses and local yielding. This novel result holds promise for practical applications, being non-destructive, controllable and isotropic [72,73,74]. It is noted, however, that, the authors have recently reported that the effect of cryogenic rejuvenation decays over time, likening the rejuvenation process to anelastic strain accumulation [75]. 

**Figure 12:**
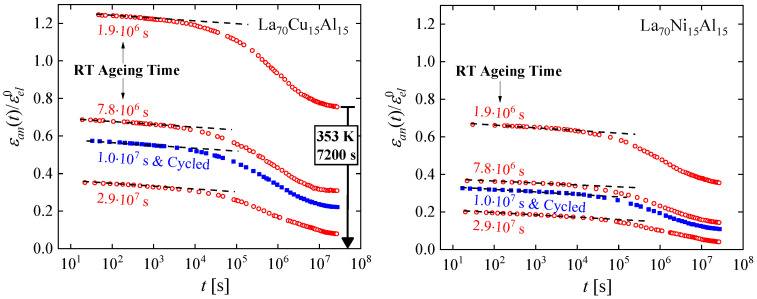
Normalized anelastic strain of La_70_Cu_15_Al_15_ and La_70_Ni_15_Al_15_ as a function of time for different aging times prior to bending, as indicated. Open circles and filled squares correspond, respectively, to measurements without and with cryogenic cycling after aging, prior to bending. Curves are not shifted. The dashed lines are all drawn with the same slope. Note that the entire strain is anelastic, as verified by annealing above room temperature (bold arrow). Reprinted from Lei, T.J.; DaCosta, L.R.; Liu, M.; Wang, W.H.; Sun Y.H.; Greer, A.L.; M. Atzmon. Microscopic characterization of structural relaxation and cryogenic rejuvenation in metallic glasses. *Acta Mater.* **2019**, 164, 165–170. Copyright (2018), with permission from Elsevier [63].

As with other examples, the lack of a periodic structure and microscopic structural probes poses challenges to obtaining a detailed description of the atomic-scale effect of cryogenic cycling. Relaxation-time spectra offer an opportunity for progress toward this goal. The nondestructive nature of cryogenic cycling offers an advantage in that the process preserves sample geometry. Two metallic glasses that undergo significant anelastic relaxation at room temperature, La_70_Cu_15_Al_15_ and La_70_Ni_15_Al_15_, were investigated [63]. Figure 12 shows the anelastic strain as a function of time after constraining and releasing the samples, following the same protocol as above. Curves were obtained for samples that were allowed to age and structurally relax for several durations, 1.9 × 10^6^ to 2.9 × 10^7^ s, prior to constraining them. In one intermediate case, samples were also cycled between room and liquid-nitrogen temperature following the aging step. Not surprisingly, the amount of anelastic strain developed during the constraining period decreased with prior room-temperature aging. For the as-prepared La_70_Cu_15_Al_15_ alloy, the anelastic strain was higher than the elastic strain at the end of the constraining period. That the stress-free strain was entirely anelastic was verified by annealing above room temperature, which resulted in a complete recovery of the original sample shape before it was constrained (See Figure 12).

**Figure 13:**
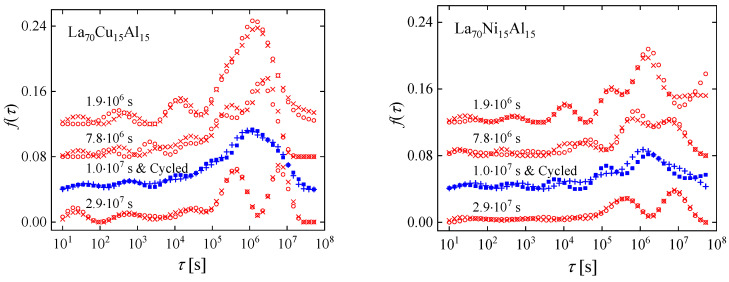
Relaxation-time spectra for La_70_Cu_15_Al_15_ and La_70_Ni_15_Al_15_ with different aging times, as indicated. For each condition, representative data for two independent samples are shown. Open circles and crosses, vs. filled squares and pluses, correspond to samples without, vs. with, cryogenic cycling, respectively. The curves are shifted vertically for clarity. Reprinted from Lei, T.J.; DaCosta, L.R.; Liu, M.; Wang, W.H.; Sun Y.H.; Greer, A.L.; M. Atzmon. Microscopic characterization of structural relaxation and cryogenic rejuvenation in metallic glasses. *Acta Mater.* **2019**, 164, 165–170. Copyright (2018), with permission from Elsevier [63].

**Figure 14:**
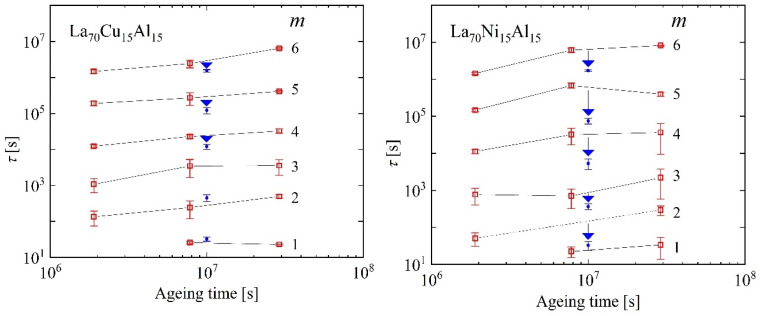
The evolution of time constants of different STZ types, *m*, with aging time for La_70_Cu_15_Al_15_ and La_70_Ni_15_Al_15_ metallic glasses. Downwards arrows indicate the effect of cryogenic cycling following aging. Reprinted from Lei, T.J.; DaCosta, L.R.; Liu, M.; Wang, W.H.; Sun Y.H.; Greer, A.L.; M. Atzmon. Microscopic characterization of structural relaxation and cryogenic rejuvenation in metallic glasses. *Acta Mater.* **2019**, 164, 165–170. Copyright (2018), with permission from Elsevier [63].

**Figure 15:**
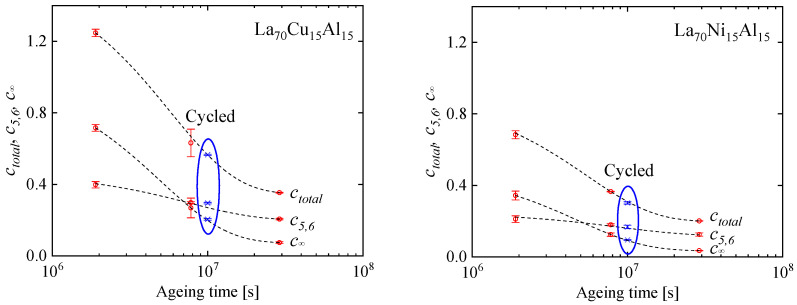
*c_∞_* = the additive term in the spectrum fit, *c_5,6_* = the integrated area of the last two peaks, and *c_total_ =* the integrated area of the entire spectrum plus *c_∞_* vs. aging time for La_70_Cu_15_Al_15_ and La_70_Ni_15_Al_15_ MGs. Blue: cycled after aging. Lines: guide to the eye. Reprinted from Lei, T.J.; DaCosta, L.R.; Liu, M.; Wang, W.H.; Sun Y.H.; Greer, A.L.; M. Atzmon. Microscopic characterization of structural relaxation and cryogenic rejuvenation in metallic glasses. *Acta Mater.* **2019**, 164, 165–170. Copyright (2018), with permission from Elsevier [63].

**Figure 16:**
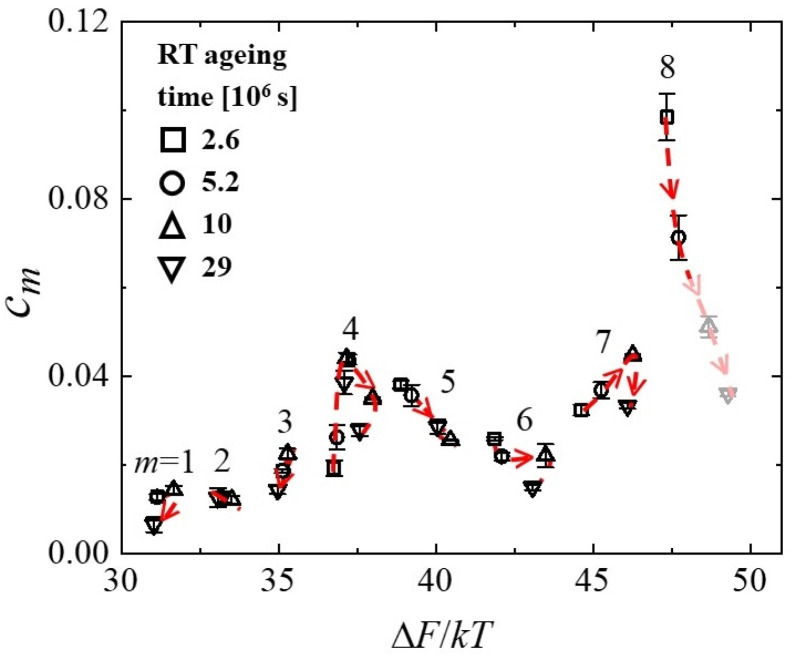
Volume fraction occupied by *m*-type *potential* STZs for La_55_Ni_20_Al_25_ metallic glass, Equation (6), as a function of activation free energy Δ*F_m_*, Equation (2), divided by *kT*, for different room-temperature aging times. Each symbol corresponds to one aging-time value. Arrows show the direction of evolution with room-temperature aging time for each *m*. *m* = 6–8 and beyond (not active at room temperature within the time range used) correspond to the α relaxation, and *m* ≤ 5 correspond to the β relaxation. The last two data points for *m* = 8 STZs represent an underestimate due to lack of mechanical equilibration at the end of the constraining period for samples with long aging time and associated long *τ*_8_ values (see discussion). Reproduced from Lei, T.J.; Liu, M.; Wang, W.H.; Sun, Y. H.; Greer, A. L.; Atzmon, M. Shear transformation zone analysis of anelastic relaxation of a metallic glass reveals distinct properties of α and β relaxations. *Phys Rev. E* **2019**, 100, 033001 [76].

In the strain curves, there is no immediately obvious effect of cryogenic cycling. However, the spectra computed from them (Figure 13) reveal further details. Similar to Al_86.8_Ni_3.7_Y_9.5_ (Figure 3), they contain distinct peaks. Room-temperature aging leads to a decrease in the intensities of the peaks, especially that of the peak with the longest time constant, and their shift to longer time constants. Interestingly, cryogenic cycling reduces the corresponding time constants (Figure 14), restoring them to pre-aging values. However, the peak intensities are not affected by cryogenic cycling: the areas under resolvable peaks or peak sets for the cycled samples fit on the same curve, as a function of aging time, as those for the aged samples that were not cycled (Figure 15). Based on the discussion in Section 4, we conclude that structural relaxation associated with aging leads to a reduction in the number of *potential* STZs. The increase in time constants is likely due to an increase in the modulus of the glass, which increases the activation free energy for shear transformations (see present Equation (2) and Figure 7 in Ref. [76]). Cryogenic rejuvenation likely restores the elastic modulus. However, it does not lead to a recovery of the number density of *potential* STZs. The impact of structural relaxation on the number density of *potential* STZs, as seen in the amount of normalized anelastic strain, is mainly on those consisting of a larger number of atoms, which are the slowest. This is seen qualitatively in Ref. [63], and in further detail for La_55_Ni_20_Al_25_ in Figure 16 [76] which shows the evolution of each *c_m_* with aging time. The decrease of *c_m_* with aging is likely a result of a decrease in free volume [12,27], as the density is known to increase with structural relaxation.

Using a wide range of experimental techniques, including x-ray photon spectroscopy, Gallino et al. [77] observed rapid relaxation, followed by quasi-stationary states in a Au-based glass. The authors describe these states as reflecting structural relaxation pathways that are decoupled from the α relaxation, not involving densification. In a related publication [78], it is shown that the vitrification kinetics upon cooling do not follow the α-relaxation kinetics. The authors attribute the multiple time scales to the existence of spatially heterogeneous events, e.g., STZs. The discussion below of interdiffusion in an amorphous multilayer (Section 12) points to a similar observation and interpretation. The detailed behavior of specific multicomponent alloys is likely to vary, depending on the mobilities of individual alloying elements. 

A few conclusions are noted here:

(1) Although cryogenic rejuvenation does not restore the *c_m_*, plasticity is improved by this process because of the increased fraction of *potential* STZ with a sufficiently short time constant to participate in deformation.
(2) A comparison of the time scale for *structural* relaxation, > 10^6^ s, with the shorter times for *anelastic* relaxation indicates that the mechanisms underlying the two processes cannot be assumed to be the same. The driving force for the former is thermodynamic, whereas for the latter it is mechanical.
(3) While a measurement of a single variable, e.g., stored enthalpy or plasticity, may give the impression that the cryogenic cycling process leads to a reversal of structural relaxation due to aging, these results clearly show that the details are more nuanced. Generally, structural relaxation and rejuvenation cannot be described with a single variable.

## 10. The Mechanism of the β Relaxation [76] 

Many glasses and glass types exhibit high-frequency secondary (β) relaxations in their dynamic response [79,80,81,82]. These manifest in a second peak or a tail, e.g., in their loss modulus or dielectric susceptibility. For molecular glasses, the corresponding mechanisms can be straightforward – intermolecular vs. intramolecular relaxations. No such obvious distinction is known for metallic glasses. Nevertheless, it has been suggested that β relaxations are due to a separate mechanism, further correlating it with plasticity and adding speculations on its atomistic details [83,84]. Higher-frequency (γ) relaxations have also been reported [85]. Our experimental results and their analysis can be used to evaluate this approach. In this section, the atomic-scale mechanism is discussed, and the correlation between α and β relaxations and plasticity will be evaluated in the next section.

**Figure 17:**
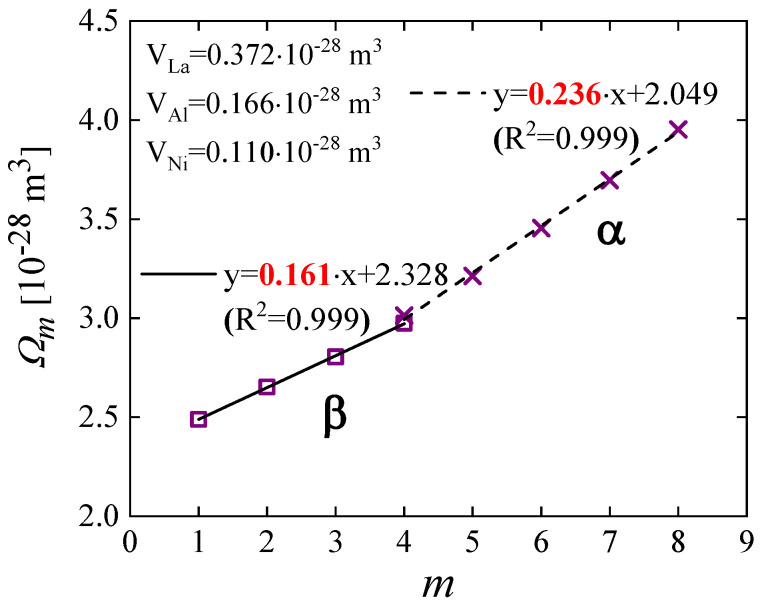
La_55_Ni_20_Al_25_: STZ volume (*Ω_m_*) as a function STZ type (*m*) for samples aged 2.9 × 10^7^ s. The error bars, <0.7%, are smaller than the symbols. The slopes correspond to the volume increment between two adjacent *Ω_m_* values. The random error in these slopes is 2–3%. Reproduced from Lei, T.J.; Liu, M.; Wang, W.H.; Sun, Y. H.; Greer, A. L.; Atzmon, M. Shear transformation zone analysis of anelastic relaxation of a metallic glass reveals distinct properties of α and β relaxations. *Phys Rev. E* **2019**, 100, 033001 [76].

Equipped with a new methodology of analyzing mechanical relaxations [18], we studied the STZ spectra of La_55_Ni_20_Al_25_, a metallic glass with significant β relaxation [76]. A plot of the STZ volume, $\Omega_{m}$, as a function of *m*, Figure 17, reveals two regimes. The atomic volume obtained from the slope is 0.161 × 10^−28^ m^3^ for small and fast STZs, and 0.236 × 10^−28^ m^3^ for large and slow STZs. As before, the straight-line fits are excellent. The former value is close to that of an Al atom, 0.166 × 10^−28^ m^3^, whereas the latter is within about 12% of the mean atomic volume of the alloy. Similar results, with two slope regimes, were observed in La_70_Cu_15_Al_15_ and La_70_Ni_15_Al_15_ [86]. While it may be speculative to take these slope values literally, they suggest that different elements play a role in fast vs. slow STZs.

In Refs. [18,76,86], a single mechanism, namely shear transformations, consistently describes the entire range of relaxation times observed. This suggests that even though the β relaxation appears distinct in the loss modulus for some metallic glasses, a separate mechanism need not be invoked. This is seen when *E*″(*ω*) is computed [44] from the experimental spectrum obtained for quasi-static relaxation [18] – it exhibits a high-frequency tail (Figure 9b) despite the fact that the spectrum corresponds to a single mechanism.

In much of the literature, e.g., Refs. [87,88], the α and β relaxations are discussed in terms of their reversibility vs. irreversibility. The α relaxation, generally associated with the glass transition, is described as irreversible whereas the β relaxation is described as reversible. Ref. [89] goes further and suggests that some β relaxations are reversible, and others are not. We argue that reversibility or lack thereof are not inherent properties of these relaxations. As mentioned above, STZs are reversible when their volume fraction is small, but become irreversible at high volume fraction as a result of loss of back-stress. Our anelastic relaxation experiments demonstrate that all STZ sizes, including those underlying both the α and β relaxation, are reversible at small strains, when they occupy a small volume fraction. 

## 11. STZ Properties and Plasticity [86]

**Figure 18:**
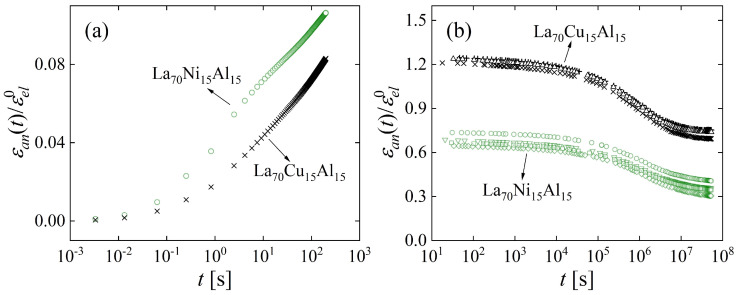
Anelastic strain, normalized by the corresponding equilibrium elastic strain, vs. time for (**a**) cantilever bending and (**b**) mandrel measurements for La_70_Cu_15_Al_15_ and La_70_Ni_15_Al_15_ with a prior room-temperature aging time of 1.9 × 10^6^ s. For cantilever bending, an average of all tests for the same composition is displayed, and each point is an average of 500 experimental data points. For the mandrel measurements, data corresponding to all samples are shown. Reprinted from Lei, T.J.; DaCosta, L. R.; Liu, M.; Shen, J.; Sun, Y. H.; Wang, W.H.; Atzmon, M. Composition dependence of metallic glass plasticity and its prediction from anelastic relaxation–A shear transformation zone analysis. *Acta Mater. ***2020**, *195*, 81–86, Copyright (2020), with permission from Elsevier [86].

**Figure 19:**
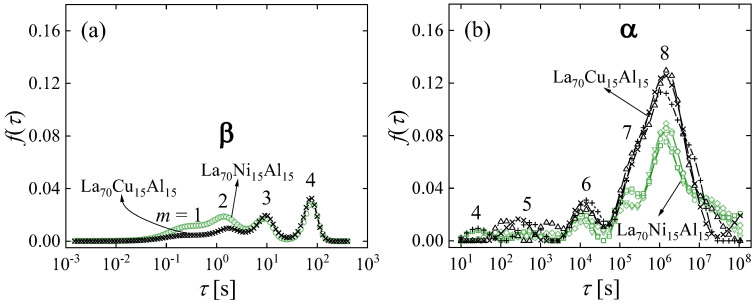
Relaxation-time spectra for (**a**) cantilever bending and (**b**) mandrel measurements, computed from the normalized anelastic strain vs. time data of Figure 18 for La_70_Cu_15_Al_15_ and La_70_Ni_15_Al_15_ aged at room temperature for 1.9 × 10^6^ s. For cantilever bending, an average of all spectra is shown for each alloy, and the standard deviation of the mean is smaller than the symbols. All spectra are included for the mandrel measurements. Peaks are numbered *m* = 1,…,8, corresponding to different STZ types. Reprinted from Lei, T.J.; DaCosta, L. R.; Liu, M.; Shen, J.; Sun, Y. H.; Wang, W.H.; Atzmon, M. Composition dependence of metallic glass plasticity and its prediction from anelastic relaxation–A shear transformation zone analysis. *Acta Mater.* **2020**, *195*, 81–86, Copyright (2020), with permission from Elsevier [86].

As mentioned above, it has been suggested that the β relaxation is responsible for alloy plasticity [83,84]. One observation that inspired this assertion was the similarity in activation energy for plasticity and the β relaxation. An opportunity for a deeper evaluation of the origin of plasticity presents itself in the alloys La_70_Cu_15_Al_15_ and La_70_Ni_15_Al_15_. Despite their similar compositions, the latter exhibits an intense β relaxation, whereas the former only exhibits a shoulder in the loss modulus, *E″*(*ω*) [90]. The same methodology was used as in the cases above to determine the STZ spectra, followed by comparison with the tensile behavior. The normalized strain evolution (Figure 18) and the corresponding spectra (Figure 19) show that La_70_Ni_15_Al_15_ contains a higher fraction of fast STZs, which correspond to β relaxations, than La_70_Cu_15_Al_15_, whereas the opposite is true for slow STZs, which correspond to α relaxations. This agrees qualitatively with *E″*(*ω*) data [90]. It should be noted that loss-modulus data are typically normalized by the α peak, so the β intensity observed in *E″*(*ω*) is relative. In contrast, the present spectrum peak areas provide *absolute* information on the volume fraction occupied by STZs. One therefore finds that when *E″*(*ω*) is normalized, a lower α intensity for La_70_Ni_15_Al_15_ further enhances its apparent β intensity, as compared with La_70_Cu_15_Al_15_.

**Figure 20:**
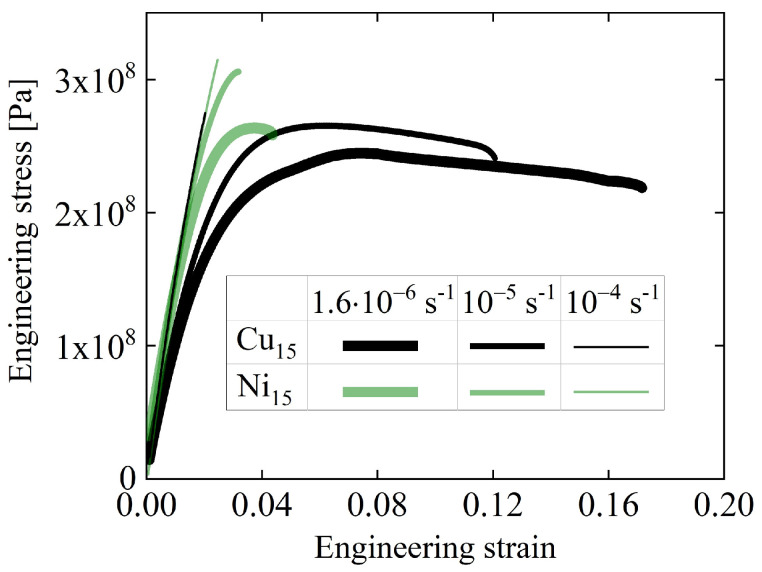
Engineering stress vs. engineering strain for La_70_Cu_15_Al_15_ and La_70_Ni_15_Al_15_ obtained from room-temperature tensile tests at strain rates of 1.6 × 10^−6^ s^−1^, 10^−5^ s^−1^, and 10^−4^ s^−1^. Curve thickness decreases with increasing strain rates. Each curve consists of 200–20,000 data points, depending on rate. Reprinted from Lei, T.J.; DaCosta, L. R.; Liu, M.; Shen, J.; Sun, Y. H.; Wang, W.H.; Atzmon, M. Composition dependence of metallic glass plasticity and its prediction from anelastic relaxation–A shear transformation zone analysis. *Acta Mater.* **2020**, *195*, 81–86, Copyright (2020), with permission from Elsevier [86].

The tensile behavior of the two alloys was compared at engineering strain rates of 1.6 × 10^−6^ s^−1^ to 10^−4^ s^−1^ (Figure 20). At the two lower rates, La_70_Cu_15_Al_15_ exhibited far greater plasticity, with up to > 17% engineering strain. It is noted that this example shows a negative correlation of β intensity with plasticity, the opposite of that proposed in Ref. [83]. While 17% engineering strain is beyond the linear regime of non-interacting STZs, the STZ spectra can be used to qualitatively explain the difference in mechanical behavior between the alloys. 

For the stress applied in the tensile measurements, the strain rate obtained is higher than that expected from the active STZs observed. Similarly, the total strain in Figure 20 cannot be accounted for by the total volume fraction of active STZs contributing to Figure 17 and Figure 18. This is likely a result of STZ interactions due to their high concentration at these strain values, leading to a reduction in their elastic energy barrier and therefore enhanced kinetics [19]. Thus, larger STZs than those participating in linear anelasticity contribute to the observed room-temperature tensile deformation. We propose that the deformation observed in Figure 20 does not represent an activated flow state, such as described in Ref. [12], for which a steady-state structure is achieved by repeated regeneration. Rather, we argue, this deformation is in a transient state, which persists as long as *potential* STZs keep up with the applied strain. Beyond this point, the increasing applied strain is accommodated by localization, resulting in failure. Since the La_70_Cu_15_Al_15_ alloy contains an *overall* higher volume fraction occupied by *potential* STZs than La_70_Ni_15_Al_15_ does, the former reaches higher strains before it fails. 

## 12. Additional Properties 

There may be potential for expanding the STZ model to describe additional atomic transport phenomena not addressed in this review. Argon and Shi [19] address the limit of the model, when back-stress is lost due to STZs being in close proximity to each other. Short-range diffusion, e.g., in multilayered thin films with short modulation wavelength, could take place by small displacements associated with STZs. This would be consistent with the observation of two different time constants for interdiffusion in modulated Ni-Zr thin films [91]. Flow or long-range diffusion would require STZ percolation. For flow to take place, the volume fraction that is rigid, i.e., does *not* contain active *potential* STZs, $exp\left(-\sum_{n=1}^{n_{0}}c_{n}\right)$, where *n_0_* is the temperature-dependent maximum size of such *potential* STZs, has to be *below* the percolation threshold. Otherwise, the matrix is rigid. The requirement for long-range diffusion is less strict: 1 − $exp\left(-\sum_{n=1}^{n_{0}}c_{n}\right)$, the volume fraction occupied by active *potential* STZs, has to exceed the percolation threshold. As noted above, overlapping *potential* STZs are counted multiple times, so $\sum_{n=1}^{n_{0}}c_{n}>1$ is possible [27].

## 13. Conclusions 

Due to the lack of periodicity, microscopic atomic rearrangements in metallic glasses can typically only be inferred indirectly from experiment. While physical analogs and molecular dynamics have contributed important insights, they are not suitable for simulating processes with a wide range of activation free energy and therefore time constants. We show here that anelastic relaxation, conducted over a wide range of time constants, can provide important insights when combined with spectrum determination. This work will hopefully motivate further simulations and experiments. For example, since the number of directions in phase space is too large to comprehensively capture in atomistic simulations, the present results could offer possible directions to probe in order to determine the barriers to possible shear transformations, e.g., by extending Ref. [92]. Atom-probe tomography investigations of chemical heterogeneity could help evaluate the conclusions that suggest composition differences between fast and slow STZs.

## Data Availability

No new data were created or analyzed in this study. Data sharing is not applicable to this article.
